# Nonanatomic healing of the greater tuberosity after plating in proximal humeral fractures: a case control study

**DOI:** 10.1186/s13018-023-03811-8

**Published:** 2023-05-19

**Authors:** Ning Sheng, Tingwang Shi, Qiuke Wang, Lei Wang, Yunfeng Chen

**Affiliations:** Department of Orthopedic Surgery, Shanghai Jiao Tong University Affiliated Sixth People’s Hospital, 600 Yishan Road, Shanghai, 200233 People’s Republic of China

**Keywords:** Proximal humeral fractures, Plate, Greater tuberosity, Risk factors

## Abstract

**Background:**

Open reduction and plate internal fixation (ORIF) is one of the most common treatment methods for proximal humeral fractures. Complications associated with the greater tuberosity (GT) are rarely reported, therefore, the purpose of this study was to analyze the complications associated with the GT and the risk factors after locked-plate internal fixation.

**Methods:**

We retrospectively analyzed the medical and radiographic data of patients with proximal humeral fractures involving the GT treated with locking plates between January 2016 and July 2019. We divided all patients into two groups, the anatomic GT healing group and the nonanatomic GT healing group, depending on the radiographic outcomes of the GT. Clinical outcome was assessed by the Constant scoring system. Potential risk factors included preoperative and intraoperative factors. Preoperative factors included sex, age, body mass index, fracture type, fracture-dislocation, proximal humeral bone mineral density, humeral head extension, hinge integrity, comminuted GT, volume and surface area of the main GT fragment, and displacement of the main GT fragment. Intraoperative factors were adequate medial support, residual head-shaft displacement, head-shaft angle and residual GT displacement. Univariate logistic regression and multivariate logistic regression were used to identify risk factors.

**Results:**

There were 207 patients (130 women and 77 men; mean age, 55 years). GT anatomic healing was observed in 139 (67.1%) patients and nonanatomic healing in 68 (32.9%). Patients with GT nonanatomic healing had significantly inferior Constant scores than those with GT anatomic healing (75.0 ± 13.9 vs. 83.9 ± 11.8, *P* < 0.001). Patients with high GT malposition had worse Constant scores than patients with low GT malposition (73.3 ± 12.7 vs. 81.1 ± 11.4, *P* = 0.039). The multivariate logistic model showed that GT fracture characteristics were not risk factors for nonanatomic GT healing, while residual GT displacement was.

**Conclusions:**

Nonanatomic healing of the GT is a high-rate complication of proximal humeral fractures, resulting in inferior clinical outcomes, especially for high GT malposition. Fracture characteristics of the GT are not risk factors for GT nonanatomic healing and GT comminution should not be regarded as a contraindication to ORIF for proximal humeral fractures.

## Background

Proximal humeral fractures are the third most common type of fracture in older patients, accounting for 5–6% of all fractures [1, 2]. Most proximal humeral fractures are minimally displaced and treated conservatively. Displaced and unstable proximal humeral fractures are commonly treated surgically [3]. Primary open reduction and plate internal fixation (ORIF) is indicated for reconstructable proximal humeral fractures, especially in younger and more active patients with greater functional expectations [4, 5].

Research on isolated GT fractures has shown that malposition or nonunion of the GT causes variable rotator cuff weakness and results in a poor outcome [6, 7]. Levy et al. found that the abduction force was significantly increased by GT displacements of 5 mm [8]. Patients with a GT displacement of more than 3 mm have poorer results than those with less displacement [9]. GT also affects clinical outcomes after reverse shoulder arthroplasty for proximal humeral fractures [10, 11].

Many studies have reported the complications of proximal humeral fractures after plating, but the outcomes of GT are rarely mentioned [12–15]. Nonanatomic healing of the GT may be the reason why some patients with a proximal humeral fracture and “good” fracture healing have a partial shoulder function defect. There is no comprehensive analysis of the reasons for nonanatomic healing of the GT after plating in proximal humeral fractures involving the GT. This study aimed to determine the rate of nonanatomic healing of the GT and determine whether GT fracture characteristics are risk factors for GT nonanatomic healing. We hypothesized that nonanatomic healing of the GT would be a common complication in proximal humeral fractures and that residual GT displacement could be a risk factor for GT nonanatomic healing.

## Methods

### Patient selection

All trauma center patients with proximal humeral fractures treated with locking plates between January 2016 and July 2019 were retrospectively identified using the hospital information system. This clinical study was approved by the local research ethics committee (2020-KY-088(K)).

The inclusion criteria were as follows: (a) acute unilateral proximal humeral fractures involving the GT; (b) surgical treatment with ORIF; and (c) complete radiographic records including preoperative anteroposterior radiographs and computed tomography (CT) scans with a slice thickness of 1.5 mm (anteroposterior and axillary radiographs taken both on the first day and a year or more after surgery). Patients under 18 years of age; those with open, multiple, or pathologic fractures; those who underwent revision surgery; those with a previous disease history affecting shoulder function; and those who refused to participate in the study were excluded. Written informed consent was obtained from all patients included in the study, and their medical and radiographic data were collected. The Constant scoring system was used to assess clinical outcomes. Finally, 207 patients were included with an average follow-up time of 25 months (range, 12–43 months).

**Fig. 1:**
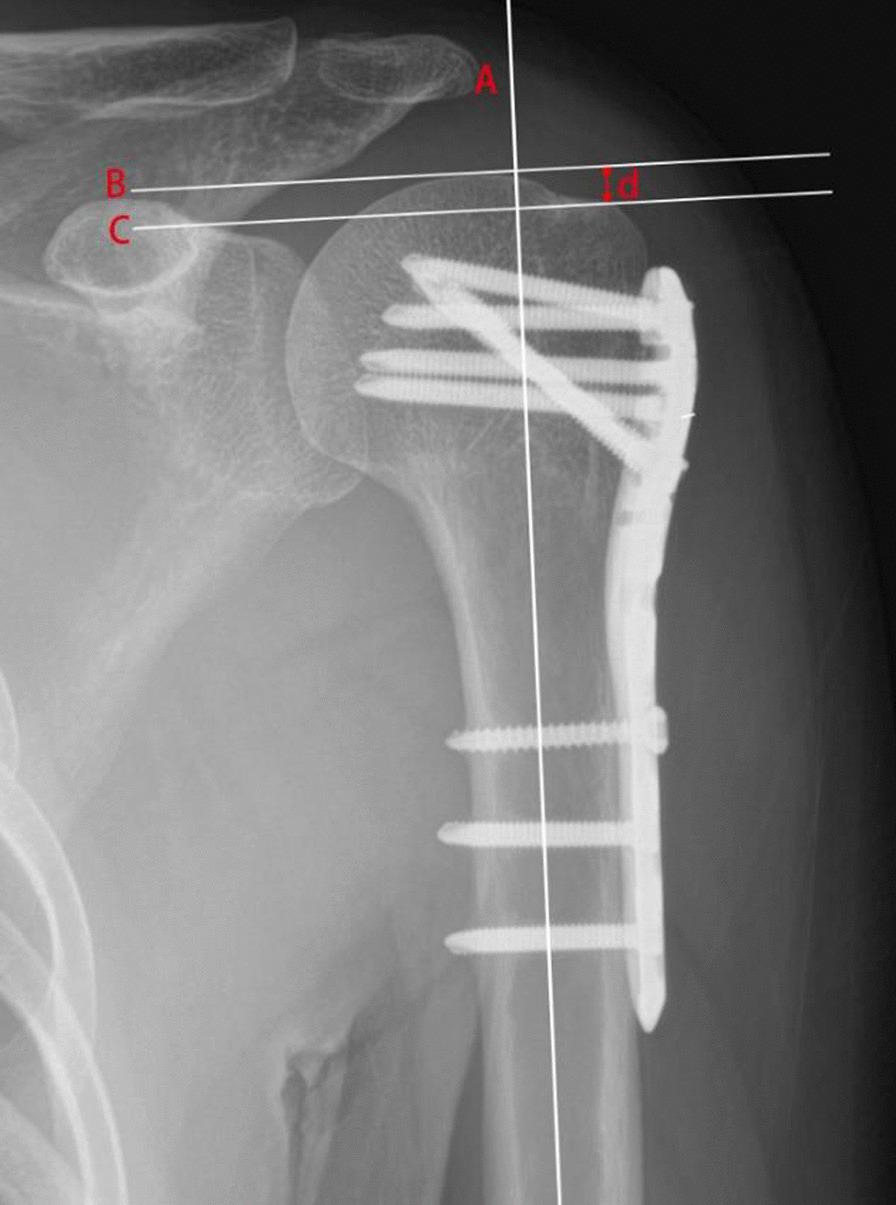
Measurement of GT position. Line A was drawn parallel to the humeral shaft. Line B and line C were drawn perpendicular to Line A, line B was placed at the upper end of the humeral head and line C was placed at the upper end of the GT. The distance (d) between line B and line C was measured. When the distance (d) was between 5 and 10 mm, the GT was considered correctly position. Malposition of the GT was defined as too low or high

**Fig. 2:**
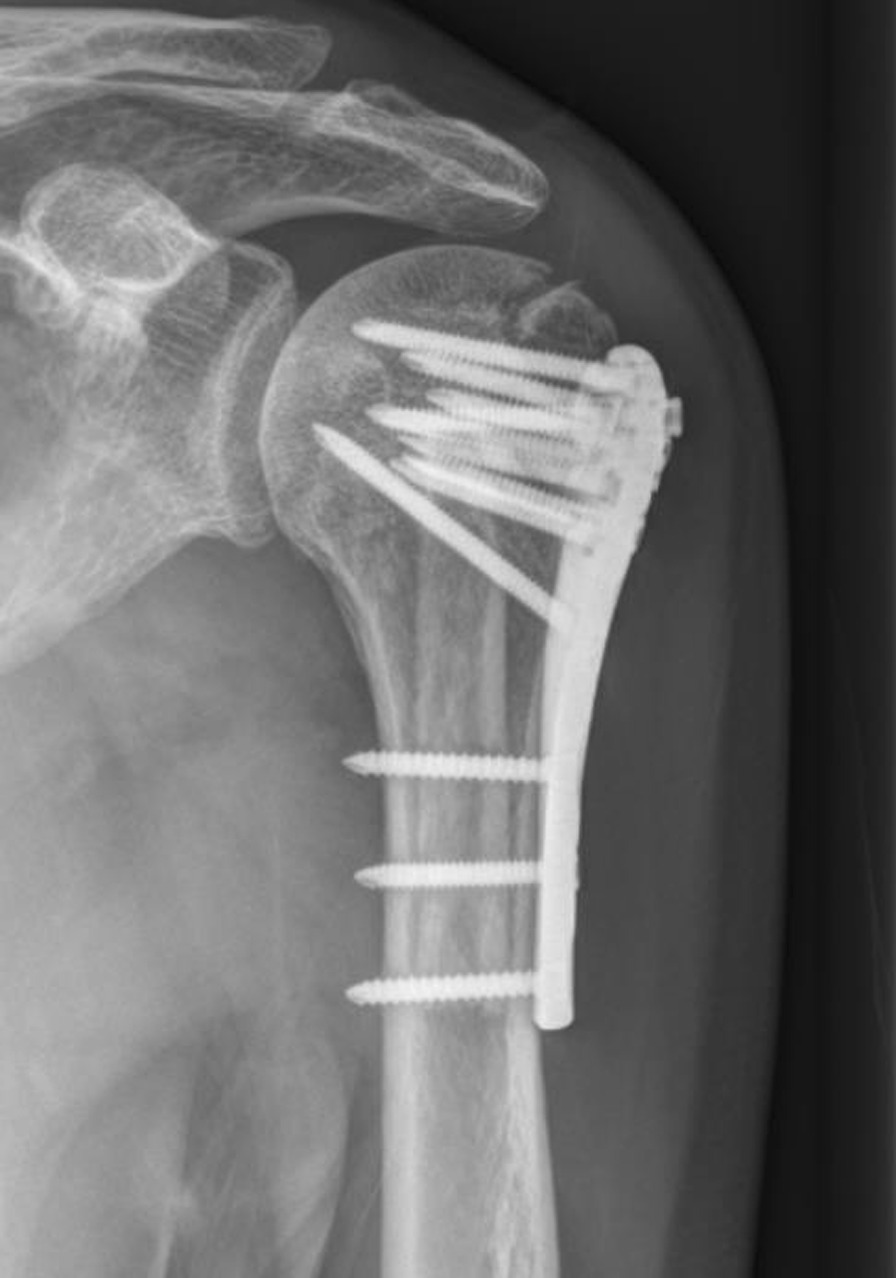
Radiograph for a patient with GT nonunion 12 months after surgery

**Fig. 3:**
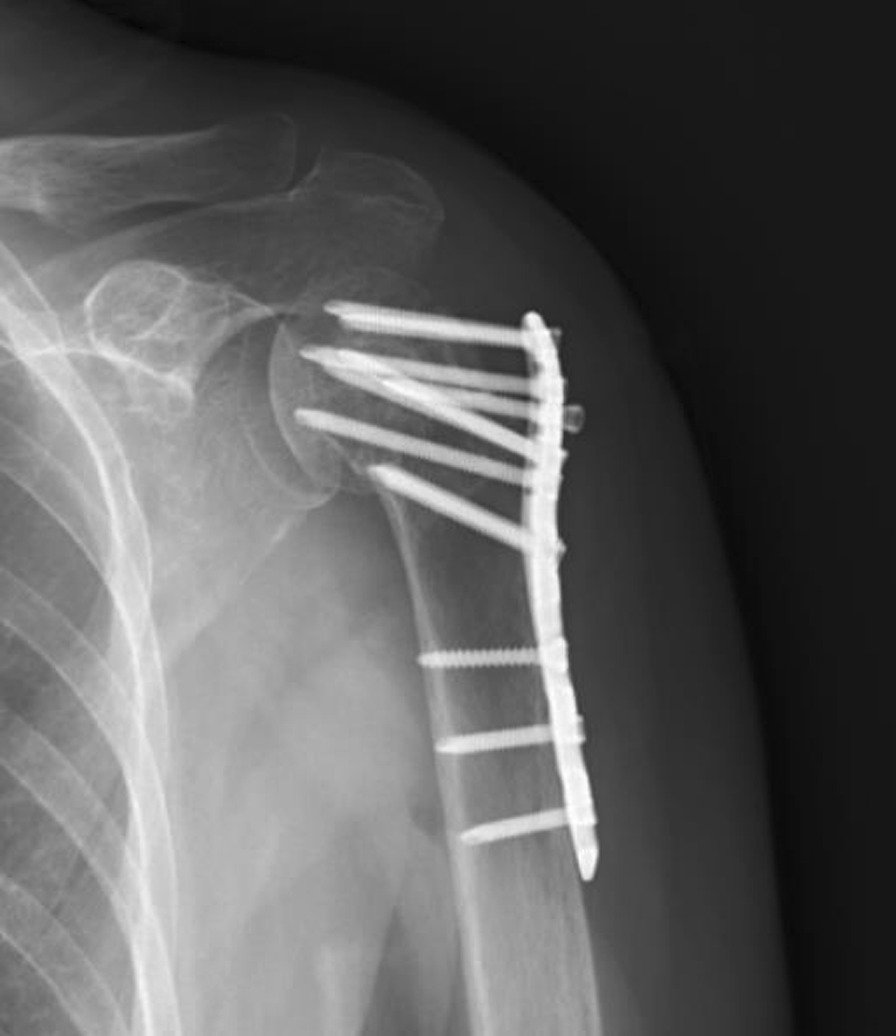
Radiograph for a patient with GT osteolysis 12 months after surgery

We divided all patients into two groups, the anatomic GT healing group (AH) and the nonanatomic GT healing group (NH), depending on the radiographic outcomes of the GT. Nonanatomic GT healing included malposition, nonunion, and osteolysis of the GT. According to previous studies, the GT was considered correctly positioned when it was visible in the anteroposterior view in neutral rotation, and its summit was between 5 and 10 mm below the summit of the head (Fig. 1). Malposition of the GT was defined as the summit of the GT being less than 5 mm or more than 10 mm below the summit of the humeral head on the anteroposterior radiograph in neutral rotation. It was defined as high when the summit of the GT was less than 5 mm below the summit of the head and as low when the summit of the GT was more than 10 mm below the summit of the head [6, 16]. Nonunion and osteolysis of the GT were identified by comparing preoperative, immediate postoperative, and outpatient follow-up radiographs (Figs. 2, 3). Anatomic GT healing was defined as the absence of the three complications mentioned above and correct positioning of the GT.

Potential risk factors for nonanatomic GT healing included preoperative and intraoperative factors. Preoperative factors included sex, age, body mass index, fracture type, fracture-dislocation, proximal humeral bone mineral density (BMD), humeral head extension, hinge integrity, comminuted GT, volume and surface area of the main GT fragment, and displacement of the main GT fragment. Three orthopedic surgeons with more than 10 years of experience in the Department of Orthopedics classified fractures according to the Neer classification system. We measured proximal humeral BMD using the proximal humeral average cortical bone thickness, and an average cortical bone thickness of less than 6 mm indicated low BMD of the proximal humerus [17]. According to Hertel’s report [18], we defined humeral head extension as part of the metaphysis that remains attached to the head and the longer the extension, the more likely the head is perfused. The condition of the medial hinge was divided into comminuted or non-comminuted. The GT was defined as comminuted if there were more than two GT fracture fragments, which were estimated based on X-ray and CT (only fragments with a volume larger than 1/4 of the GT). The volume and surface area of the main GT fragment were measured using Mimics software (Materialise NV, Leuven, Belgium), which is a three-dimensional modeling software widely used in medical research [19, 20]. Raw preoperative CT data in the axial plane were imported into Mimics software to reconstruct and isolate 3-D models of the main fragment, and then the volume and surface area were measured. Horizontal and vertical displacements of the main fragment relative to the humeral head were measured on coronal and axial CT, respectively, and the final displacement of the main fragment was the sum of the horizontal and vertical displacements.

Intraoperative factors included adequate medial support, residual head-shaft displacement, head-shaft angle, and residual GT displacement after surgery. When the disrupted medial hinges were reduced with a displacement of less than 2 mm, adequate medial support was considered restored. Fractures with comminuted hinges restored adequate medial support only if augmented with allograft bone or medial plates. Patients with complete medial hinges were also considered with adequate medial support. Residual head-shaft displacement, head-shaft angle, and residual displacement of the GT were measured based on the criteria outlined in previous studies [18, 21].

Each of the two orthopedic surgeons performed all of the above assessments independently. The average measurement by these two surgeons was used for continuous variables, and a third orthopedic surgeon made the final decision if disagreements regarding discrete variables occurred.

### Surgical technique

Sixteen well-trained orthopedic surgeons performed all the operations. We placed patients in a “beach chair” position on an operating table under general anesthesia. The fracture site was exposed using the deltopectoral approach. Nonabsorbable sutures were passed through the junction of the tuberosities and rotator cuff to promote mobilization and reduce tuberosities. An adequate fibular strut was inserted into the intramedullary canal through the fracture site and medialized toward the calcar to reduce the column in patients with a comminuted medial hinge or severe osteoporosis. Subsequently, the humeral head and shaft were reduced. Once reduction was confirmed with a C-arm X-ray machine after temporary fixation, the plate (Philos plate; DePuy Synthes, Oberdorf, Switzerland; 3.5 mm LCP proximal humerus plate, IRENE, Tianjin, China) was fixed with screws. Final confirmatory orthogonal X-rays were used to show the position of the plates and screws and fracture reduction. We used No.2 Ethibond sutures to fix the tuberosities, which passed through the junction of the rotator cuff and the tuberosities. Additional supplementary tuberosity screws were used, if necessary. After testing for shoulder activity, the wound was closed.

### Statistical analysis

Frequencies and percentages were used to describe discrete variables, and the mean and standard deviation were used to describe continuous variables. Continuous variables were first tested for normal distribution using the Kolmogorov–Smirnov test. Differences in the Constant scores between the groups were calculated using the t-test or nonparametric test. Possible risk factors for nonanatomic healing of the GT were analyzed using univariate logistic regression followed by multivariate logistic regression. The area under the curve (AUC) was calculated using the receiver operating characteristic (ROC) curve, which was used to measure the model’s discriminative ability. Factors with values of *P* < 0.1 were included in the multivariate logistic regression analysis. For significant results (*P* < 0.05) in the correlation analyses, odds ratios and 95% confidence intervals were calculated. All statistical analyses were performed using SPSS statistical software (IBM SPSS Statistics for Windows, Version 21.0. Armonk, NY, USA).

## Results

**Fig. 4:**
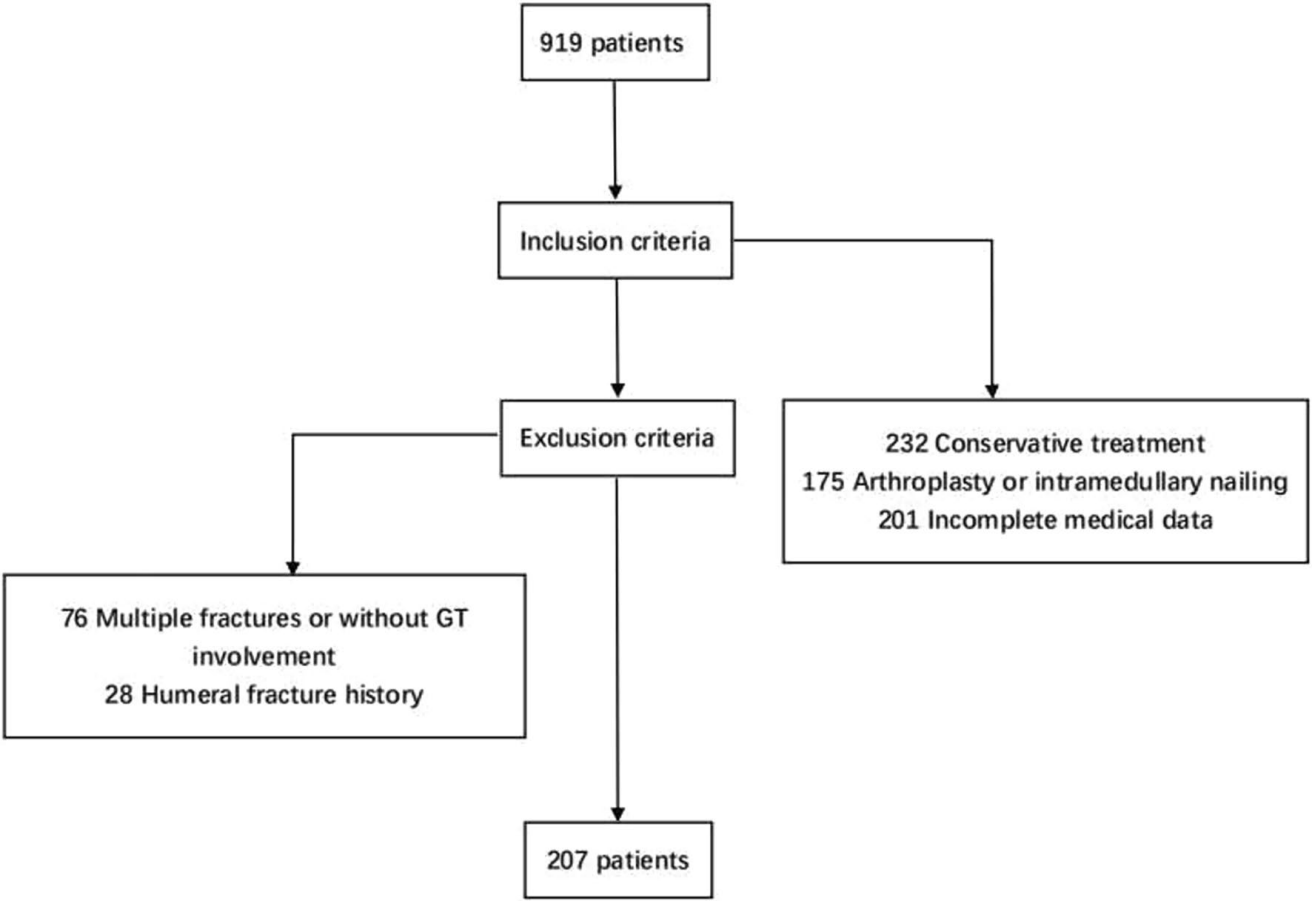
Patient selection process

A total of 919 patients with proximal humeral fractures were identified using the hospital information system from January 2016 to July 2019. Of those, 232 patients underwent conservative treatment, 175 patients were treated with arthroplasty or intramedullary nailing, and 201 patients did not have complete medical data. Only 76 patients had multiple fractures with or without GT involvement, and 28 patients had a history of humeral fracture and were excluded. The details of the inclusion process are shown in Fig. 4. Finally, 207 patients were included: 130 women (62.8%) and 77 men (37.2%) with a mean age of 55 ± 14 years (range, 19–88 years). Sixty-three (30.4%) patients had two-part proximal humeral fractures, 91 (44.0%) patients had three-part proximal humeral fractures, and 53 (25.6%) patients had four-part proximal humeral fractures. Five patients had additional lesser tuberosity screws, and none of the patients had additional supplementary GT screws. There was a total of 45 patients (21.7%) using a fibular graft, including 33 patients (15.9%) who had a comminuted medial hinge augmented with a fibular graft and 12 patients (5.8%) who had disrupted medial hinges augmented with a fibular graft for severe osteoporosis. Patient demographics and risk factors are shown in Table 1.

**Table 1:** Patient demographics and risk factors

Variable	Total (*n* = 207) *n* (%)/mean (SD)	AH (*n* = 139) *n* (%)/mean (SD)	NH (*n* = 68) *n* (%)/mean (SD)
Sex			
Female	130 (62.8%)	88 (63.3%)	42 (61.8%)
Male	77 (37.2%)	51 (36.7%)	26 (38.2%)
Age (years)	55 (± 14)	53 (± 14)	57 (± 13)
BMI	24.1 (± 3.2)	24.0 (± 3.3)	24.5 (± 2.9)
NEER classification			
Two-part	63 (30.4%)	51 (36.7%)	12 (17.6%)
Three-part	91 (44.0%)	58 (41.7%)	33 (48.5%)
Four-part	53 (25.6%)	30 (21.6%)	23 (33.8%)
Fracture-dislocation			
No	179 (86.5%)	122 (87.8%)	57 (83.8%)
Yes	28 (13.5%)	17 (12.2%)	11 (16.2%)
Low BMD of proximal humerus			
No	88 (42.5%)	62 (44.6%)	26 (38.2%)
Yes	119 (57.5%)	77 (55.4%)	42 (61.8%)
Humeral head extension			
≥ 8 mm	112 (54.1%)	86 (61.9%)	26 (38.2%)
< 8 mm	95 (45.9%)	53 (38.1%)	42 (61.8%)
Medial hinge comminuted			
No	174 (84.1%)	127 (91.4%)	47 (69.1%)
Yes	33 (15.9%)	12 (8.6%)	21 (30.9%)
GT comminuted			
No	184 (88.9%)	128 (92.1%)	56 (82.4%)
Yes	23 (11.1%)	11 (7.9%)	12 (17.6%)
Volume of the main GT fragment (mm^3^)	2180 (± 796)	2185 (± 841)	2169 (± 700)
Surface area of the main GT fragment (mm^2^)	2339 (± 689)	2334 (± 709)	2348 (± 650)
The main GT fragment separate (from humeral head)			
No	56 (27.1%)	44 (31.7%)	12 (17.6%)
Yes	151 (72.9%)	95 (68.3%)	56 (82.4%)
Displacement of the main GT fragment (mm)	7.2 (± 7.5)	6.8 (± 7.2)	8.1 (± 7.9)
Adequate medial support			
No	44 (21.3%)	27 (19.4%)	17 (25.0%)
Yes	163 (78.7%)	112 (80.6%)	51 (75.0%)
Residual head-shaft displacement			
≤ 5 mm	171 (82.6%)	116 (83.5%)	55 (80.9%)
> 5 mm	36 (17.4%)	23 (16.5%)	13 (19.1%)
Head-shaft angle			
110°–150°	182(87.9%)	120 (86.3%)	62 (91.2%)
> 150° or < 110°	25 (12.1%)	19 (13.7%)	6 (8.8%)
Residual GT displacement			
≤ 5 mm	154 (74.4%)	122 (87.8%)	32 (47.1%)
> 5 mm	53 (25.6%)	17 (12.2%)	36 (52.9%)

### Logistic regression

Out of all the 207 patients, anatomic healing of the GT was observed in 139 (67.1%) patients. A total of 68 (32.9%) patients had nonanatomic healing of the GT, with GT nonunion in 7 (3.4%), GT osteolysis in 8 (3.9%), and GT malposition in 53 (25.6%). Univariate logistic regression demonstrated that age, Neer classification, humeral head extension, condition of the medial hinge, comminuted GT, and residual GT displacement were risk factors for nonanatomic healing of the GT (Table 2). Multivariate logistic regression was performed to analyze the above eight risk factors and showed that only residual GT displacement was an independent risk factor for nonanatomic healing of the GT (Table 3). A ROC curve of only residual GT displacement was drawn, and the AUC was 0.704, indicating “fair” discriminative ability.

**Table 2:** Univariate logistic regression of the risk factors

Variable	OR	95% CI	*P* value
Sex			
Female	Reference	Reference	
Male	1.068	0.587–1.943	0.829
Age (years)	1.023	1.001–1.046	**0.041**
BMI	1.056	0.962–1.158	0.253
NEER classification			**0.016**
Two-part	Reference	Reference	
Three-part	2.418	1.131–5.172	**0.023**
Four-part	3.258	1.419–7.480	**0.005**
Fracture-dislocation			
No	Reference	Reference	
Yes	1.385	0.609–3.148	0.437
Low BMD of proximal humerus			
No	Reference	Reference	
Yes	1.301	0.719–2.352	0.384
Humeral head extension			
≥ 8 mm	Reference	Reference	
> 8 mm	2.621	1.443–4.762	**0.002**
Medial hinge comminuted			
No	Reference	Reference	
Yes	4.729	2.159–10.359	** < 0.001**
GT comminuted			
No	Reference	Reference	
Yes	2.494	1.038–5.990	**0.041**
Volume of the main GT fragment (mm^3^)	1.0	1.0–1.0	0.894
Surface area of the main GT fragment (mm^2^)	1.0	1.0–1.0	0.890
The main GT fragment separate (from humeral head)			
No	Reference	Reference	
Yes	1.505	0.774–2.926	0.228
Displacement of the main GT fragment (mm)	1.023	0.985–1.063	0.235
Adequate medial support			
Yes	Reference	Reference	
No	0.723	0.362–1.444	0.358
Residual head-shaft displacement			
≤ 5 mm	Reference	Reference	
> 5 mm	1.192	0.562–2.529	1.647
Head-shaft angle			0.328
110°–150°	Reference	Reference	
> 150° or < 110°	0.611	0.232–1.609	0.319
Residual GT displacement			0.165
≤ 5 mm	Reference	Reference	
> 5 mm	8.074	4.026–16.191	** < 0.001**

Bold indicates that the *P* value is < 0.05 and statistically significant

**Table 3:** Multivariate logistic regression of six risk factors

Variable	OR	95% CI	*P* value
Age	1.008	0.980–1.035	0.552
NEER classification			0.572
2	Reference	Reference	
3	1.637	0.642–4.174	0.302
4	1.293	0.451–3.704	0.632
Humeral head extension			
≥ 8 mm	Reference	Reference	
< 8 mm	2.138	0.959–4.769	0.063
Medial hinge comminuted			
No	Reference	Reference	
Yes	1.857	0.676–5.104	0.230
GT comminuted			
No	Reference	Reference	
Yes	1.948	0.659–5.760	0.228
Residual GT displacement			
≤ 5 mm	Reference	Reference	
> 5 mm	7.297	3.406–15.635	** < 0.001**

Bold indicates that the *P* value is < 0.05 and statistically significant

### Clinical outcomes

A total of 162 patients (101 in the AH group and 61 in the NH group) returned for clinical examination with a mean Constant score of 80.5. The average follow-up time was 26 ± 7 months (range, 12–43 months). The Constant scores in patients with nonanatomic healing of the GT were significantly different from those with anatomic healing of the GT (75.0 ± 13.9 vs. 83.9 ± 11.8, *P* < 0.001). The Constant score was 76.3 ± 12.6 in patients with GT malposition, 73.6 ± 18.1 in those with GT nonunion, and 68.3 ± 16.6 in those with GT osteolysis. There were no significant differences in the Constant scores among the three groups (*P* = 0.396) (Table 4). Among the 46 patients with GT malposition, 18 patients with malposition of the GT were defined as low, and 28 patients were defined as high. There were significant differences between the Constant scores of patients with high GT malposition and low GT malposition, (73.3 ± 12.7 vs. 81.1 ± 11.4, *P* = 0.039).

**Table 4:** Clinical outcomes of 162 patients with clinical examination

Variable	AH (*n* = 101) *n* (%)/mean (SD)	NH (*n* = 61) *n* (%)/mean (SD)	*P* value
Average follow-up time	25.5 (± 7.3)	28.1 (± 7.5)	0.280
Constant scores	83.9 (± 11.8)	75.0 (± 13.9)	** < 0.001**
GT complications			–
Malposition	–	46 (75.4%)	
Nonunion	–	7 (11.5%)	
Osteolysis	–	8 (13.1%)	
Other complications			0.053
Primary screw penetration	1 (1.0%)	1 (1.6%)	
Subsequent screw penetration	6 (5.9%)	5 (8.2%)	
Humeral head osteonecrosis^a^	3 (3.0%)	3 (4.9%)	
Heterotopic ossification^b^	2 (2.0%)	1 (1.6%)	
Posttraumatic osteoarthrosis	0	3 (4.9%)	

Bold indicates that the *P* value is < 0.05 and statistically significant

^a^Patients with humeral head osteonecrosis both with screw penetration

^b^One patient with both humeral head osteonecrosis and heterotopic ossification

### Other complications

Two patients had primary screw penetration. The final radiography showed that 11 (5.3%) patients developed subsequent screw penetration, and 6 (2.9%) of them developed subsequent humeral head osteonecrosis. Four of these patients underwent implant removal or arthroplasty, and two patients declined surgery. Three patients (1.4%) developed heterotopic ossification. Posttraumatic osteoarthrosis occurred in three patients (1.4%).

## Discussion

Our study showed a high rate of nonanatomic healing in patients with GT involving proximal humeral fractures treated with ORIF. Patients with nonanatomic healing of the GT had an inferior Constant score compared to patients with anatomic healing. It was also found that residual GT displacement is a risk factor for nonanatomic healing of the GT, rather than fracture characteristics.

Due to differences in study design, patient populations, and the surgeon’s experience, the rate of complications after ORIF has been reported to vary between 3 and 54% [22–24]. Robinson et al. reported that 77.2% of surgically treated patients had tuberosity fractures, and tuberosity involvement predicted the need for revision surgery. The complication rates mainly included postoperative stiffness in 23.6%, fixation failure in 6.8%, and osteonecrosis or posttraumatic osteoarthrosis in 4.3% of patients [25]. Although tuberosity involvement is common in complex proximal humeral fractures, the outcome of GT after plating is usually ignored, and the complication rate is rarely reported. Our study focused on the radiographic outcomes of GT at least one year after surgery and found a high rate of GT nonanatomic healing in proximal humeral fractures involving the GT. Nonanatomic healing of the GT consisted of GT malposition in 25.6%, GT nonunion in 3.4%, and GT osteolysis in 3.9% of patients. In addition, osteonecrosis or posttraumatic osteoarthrosis was observed in 4.3% of patients, which is consistent with the report by Robinson [25].

We confirmed that patients with nonanatomic healing of the GT had inferior functional results compared to those with anatomic healing of the GT. Ohl et al. reported that in elderly patients who have undergone reverse shoulder arthroplasty for acute proximal humeral fractures, patients with GT malunion, nonunion, or osteolysis had significantly lower functional results [11]. Clavert et al. analyzed the outcomes of 44 patients with proximal humeral fractures after plating and reported that the 20.7-month Constant score in patients with malunion of the GT was 57.2 points, which was significantly worse than that of patients without malunion of the GT [6]. In our study, the Constant score of patients with nonanatomic healing of the GT was 8.9 points less than that of patients with anatomic healing of the GT. Although there were no significant differences in the Constant score among the three complications, in consideration of the small sample size, further studies are needed to confirm this result. Although Kukkonen et al. reported a minimal clinically important difference estimate of 10.4 points as the threshold for the Constant score in patients with rotator cuff tear [26], we suggest that the effect of an 8.9-point difference is not insignificant in patients after ORIF. In particular, the patients in our study had an average age of 55 years and had greater functional expectations. Surgery is performed to improve shoulder function over non-operative treatment, and the complications of GT should not be overlooked.

We also found that patients with low GT malposition had better functional results than those with high GT malposition. We believe that the elongation of the rotator cuff’s arm of force may result in better function in patients with low GT malposition than in patients with high GT malposition.

To the best of our knowledge, this is the first report on the determination of the risk factors associated with GT nonanatomic healing in proximal humeral fractures after plating, including preoperative and intraoperative factors. Hertel reported that fracture type, humeral head extension length, and medial hinge integrity were predictors of humeral head ischemia [18], which was supported by subsequent studies [27, 28]. We considered these factors as potential risk factors because of their possible effects on GT blood supply. In addition, we considered that the GT fracture characteristics, such as comminution, displacement, volume, and surface area of fracture fragments, could be potential risk factors. Univariate logistic regression showed that fracture classification, humeral head extension, and comminuted medial hinge and GT affected healing of the GT, and four-part proximal humeral fractures increased the risk of nonanatomic healing of the GT compared with three-part fractures. These findings are consistent with those of previous studies [18, 27, 28]. However, the final result showed that the quality of GT reduction independently influenced fracture healing of the GT. This may be explained by the fact that the influence of fracture characteristics could be adjusted by a good reduction. Schnetzke et al. reported that the quality of reduction influences the outcome of proximal humeral fractures [6, 21]. According to our findings, GT comminution should not be regarded as a contraindication of ORIF for proximal humeral fractures and shoulder replacement should not be selected, even with obvious displacement or small fracture fragments, because it is not a risk factor for GT nonanatomic healing. Thirty-two patients with residual GT displacement of less than 5 mm developed malpositioned GT. Loss of reduction was considered to be the main reason for the malpositioned GT, which could have resulted from the inadequate fixation of the GT or improper early mobilization. We used sutures to fix the tuberosity. Patients would receive a uniform postoperative rehabilitation protocol in the early stages, and some tolerable modifications would be made for tailored care. There may be some incompatible fixation or rehabilitation for these patients.

This study has several limitations. First, this was a non-predesigned retrospective study that introduced the risk of selection bias, and the number of patients was unmanageable. Second, the operations were performed by 16 well-trained orthopedic surgeons; however, the influence of surgeons can be covered by the quality of the reduction. Third, supplementary GT fixation with sutures was not recorded. The reason for this is that in the normal surgical method, the sutures are passed through the junction of the rotator cuff and the tuberosities to fix the tuberosities in proximal humeral fractures with tuberosity involvement. Fourth, the detailed influence of early mobilization was not included in our analysis, which may cause displacement of fragments. In our routine practice, patients would receive a uniform postoperative rehabilitation protocol in the first month after surgery. For tailored care, some modifications would be made, which, to some degree, would lead to differences. Finally, we were not able to elaborate on whether the patients have rotator cuff rupture after trauma or not, however, if a rotator cuff injury is found intraoperatively, rotator cuff reconstruction is routinely performed in our hospital.

## Conclusion

In conclusion, nonanatomic healing of the GT is a high-rate complication of proximal humeral fractures, resulting in inferior clinical outcomes, especially for high GT malposition. Fracture characteristics of the GT are not risk factors for GT nonanatomic healing and GT comminution should not be regarded as a contraindication to ORIF for proximal humeral fractures.

## Data Availability

The datasets in this study are available from the corresponding author on reasonable request.

## Abbreviations

ORIF: Open reduction and plate internal fixation
GT: Greater tuberosity
CT: Computed tomography
AH: Anatomic greater tuberosity healing
NH: Nonanatomic greater tuberosity healing
BMD: Bone mineral density
AUC: Area under the curve
ROC: Receiver operating characteristic
